# Feasibility of Tracking Human Kinematics with Simultaneous Localization and Mapping (SLAM)

**DOI:** 10.3390/s22239378

**Published:** 2022-12-01

**Authors:** Sepehr Laal, Paul Vasilyev, Sean Pearson, Mateo Aboy, James McNames

**Affiliations:** 1 Department of Electrical and Computer Engineering, Portland State University, Portland, OR 97201, USA; 2 APDM Wearable Technologies, Portland, OR 97201, USA; 3 Centre for Law, Medicine and Life Sciences, University of Cambridge, Cambridge CB2 1TN, UK

**Keywords:** computer vision, kinematics, motion capture, simultaneous localization and mapping, wearable cameras

## Abstract

We evaluated a new wearable technology that fuses inertial sensors and cameras for tracking human kinematics. These devices use on-board simultaneous localization and mapping (SLAM) algorithms to localize the camera within the environment. Significance of this technology is in its potential to overcome many of the limitations of the other dominant technologies. Our results demonstrate this system often attains an estimated orientation error of less than 1° and a position error of less than 4 cm as compared to a robotic arm. This demonstrates that SLAM’s accuracy is adequate for many practical applications for tracking human kinematics.

## 1. Introduction

Simultaneous Localization And Mapping (SLAM) is a computer vision technology that uses video from a moving camera to simultaneously determine the camera location within an unknown environment and to create a three-dimensional map of the environment [1]. Over the last 10–15 years, the accuracy and ease of use has improved due to advances in digital cameras (resolution, frame rate, optical quality, size), algorithm design, computational capacity, battery life, wireless communications, and storage [2]. The development of this technology has been driven by a variety of applications in the entertainment industry (gaming and motion picture special effects), robotics, military applications, autonomous systems development, and augmented reality [3]. The accuracy of these systems depends on many factors, but in some applications and carefully controlled conditions the positioning error can be less than 1 cm and the orientation error can be less than 1° [4].

This article discusses the viability of SLAM for tracking human kinematics with wearable cameras. In some respects this is similar to popular optical motion capture systems that use reflective markers or active light-emitting markers to track human movement. The traditional approach can be considered as an outside-in approach because the cameras are rigidly mounted in the environment focused in on limited region where the human activity is performed. SLAM uses an inside-out approach in which the cameras are worn on the body and look out at the environment at landmarks that are determined automatically [5].

SLAM offers two key potential advantages over optical motion capture systems. First, SLAM automatically identifies landmarks, which are often called anchor points, within its environment that are used to determine the camera orientation and position. This potentially enables SLAM to be used at almost any location, inside or outside, without limitations on the field of view. Second, SLAM may become available at a smaller cost to purchase and operate, since it does not require a dedicated laboratory with specialized cameras.

Wearable motion sensors with accelerometers, gyroscopes, and magnetometers can also overcome many of the limitations of optical motion capture systems, but have their own limitations. For example the magnetic field in many indoor environments is non-uniform so it is difficult to use the magnetometer in these locations. Inertial sensors also have difficulty estimating absolute position, since it can only be estimated by integrating the acceleration after correcting for the effects of gravity [6]. Some groups have also developed flexible sensors to measure the angles of joints between adjacent body segments, but they do not measure absolute position and orientation [7]. SLAM has the potential to overcome these limitations. It directly estimates the absolute position and orientation of the camera within an environment. It does not require integration and does not use the local magnetic field.

SLAM also has some limitations that may make it unsuitable for tracking human motion. The accuracy may be compromised when the cameras are occluded, the illumination is poor, the environment does not include many uniquely identifiable features, or the environment includes many moving features such as in crowds of people, water, or leaves of trees. SLAM may also have difficulty tracking rapid movements due to motion blur and automatic white balancing [8]. For applications that require tracking of joint angles, SLAM requires some form of calibration that enables the camera orientation and position to be transformed to the location of the body segment to which the camera is attached. Similar approaches to those used to align inertial sensors with body segments can be used [9]. The accuracy of the location estimates partly depends on the completeness of the map of the environment, so it may be initially less accurate at location tracking until the map has stabilized.

We are only aware of one group that has used SLAM with wearable cameras to quantify human kinematics [8]. They described a system based on 14 body-mounted GoPro cameras for estimating full body kinematics. Although the results compared to an optical motion capture system were encouraging (median joint position errors of 1.76 cm and joint angle errors of 1.81°), this initial prototype was limited by the effects of motion blur, automatic white balancing, rolling shutter effects, and motion within the scenes recorded by the cameras. These were also the combined results that included a skeletal model. The constraints of the kinematic model and combination of multiple cameras helped reduce the error. They did not report the accuracy of a single camera.

There are also many approaches to integrating information from additional sensors to improve accuracy. For example modern systems may use multiple cameras that are looking at the same feature points at the same time [10]. These systems can also integrate inertial sensors [11], depth sensors [12], and LIDAR [13] to improve accuracy further. These systems have the potential to overcome many of the individual limitations by combining the strengths of each of the underlying technologies. For example, SLAM may not be able to track rapid changes in orientation due to motion blur, but gyroscopes are well suited for tracking these rapid movements. In contrast, SLAM is exceptionally good at tracking orientation during slow movements over long periods of time, which is when gyroscopes perform poorly due to sensor drift and the accumulation of error in integrating the rotational velocity. The fusion of these two sensing technologies has the potential to perform well under both conditions [14].

SLAM hardware and algorithms have improved substantially in recent years. However, tracking human kinematics with a wearable camera is a different application with different types of camera movement than most other SLAM applications. For example, during some vigorous movements the camera may rotate rapidly. This type of movement does not tend to occur in other applications in which the camera platforms are less dynamic, as often occurs in robotics and autonomous systems. It is not known how accurate these systems are at tracking human movement. The primary aim of this paper is to determine how accurately a modern off-the-shelf SLAM system can track human movement.

## 2. Materials and Methods

This section describes the SLAM system that we selected for evaluation, as well as two different reference systems, an optical motion capture system and a robotic arm, to evaluate the performance of the SLAM system during typical human movements. This section also describes our study design, how the systems were synchronized and aligned, and the measures of performance for position and orientation.

### 2.1. SLAM Platform Selection

We selected Intel’s T265 RealSense tracking camera as a system that represents the current state of the art. This system includes two fisheye cameras with 163 ± 5° field of view, global shutters, and 848 × 800 active pixels. It also uses an inertial measurement unit to improve performance during rapid movements. This unit includes an accelerometer with a range of ± 4 g sampled at 200 Hz and a gyroscope with a range of ±2000 °/s sampled at 62.5 Hz. It is relatively compact (108.0 × 24.5 × 12.5 mm) and light weight (60 g). It consumes 2.3 W and could provide hours of operation on battery power.

The T265 camera uses a proprietary V-SLAM (Visual SLAM) algorithm that runs directly in real time on the device and is designed to support many applications including augmented and virtual reality. The algorithm uses loop closure, map storage, map export, and relocalizes the camera if the trajectory is lost [15].

### 2.2. Optical Motion Capture Lab

To evaluate the tracking performance of the T265 camera, we used an optical motion capture system manufactured by Motion Analysis, Inc. (Rohnert Park, CA, USA). The system is comprised of 8 Raptor 12 HS cameras and 4 Kestrel 300 cameras, for a total of 12 cameras. The 8 Raptor 12 HS cameras are mounted in a circular pattern near the ceiling providing them with 360 degree coverage of the lab below. The four Kestrel 300 cameras are mounted on tripods. The cameras were positioned to improve coverage volume in areas of occlusion or where higher precision tracking is desired. All cameras were configured for a maximum frame rate of 120 Hz. With proper camera calibration and under carefully controlled conditions, motion capture systems similar to ours have demonstrated sub-millimeter accuracy when tracking a 15.9 mm diameter retroreflective marker target [16].

**Figure 1:**
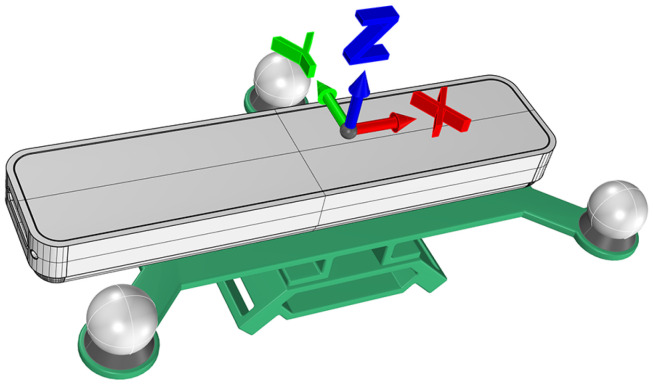
3D Model of jig with attached Intel T265 camera.

**Figure 2:**
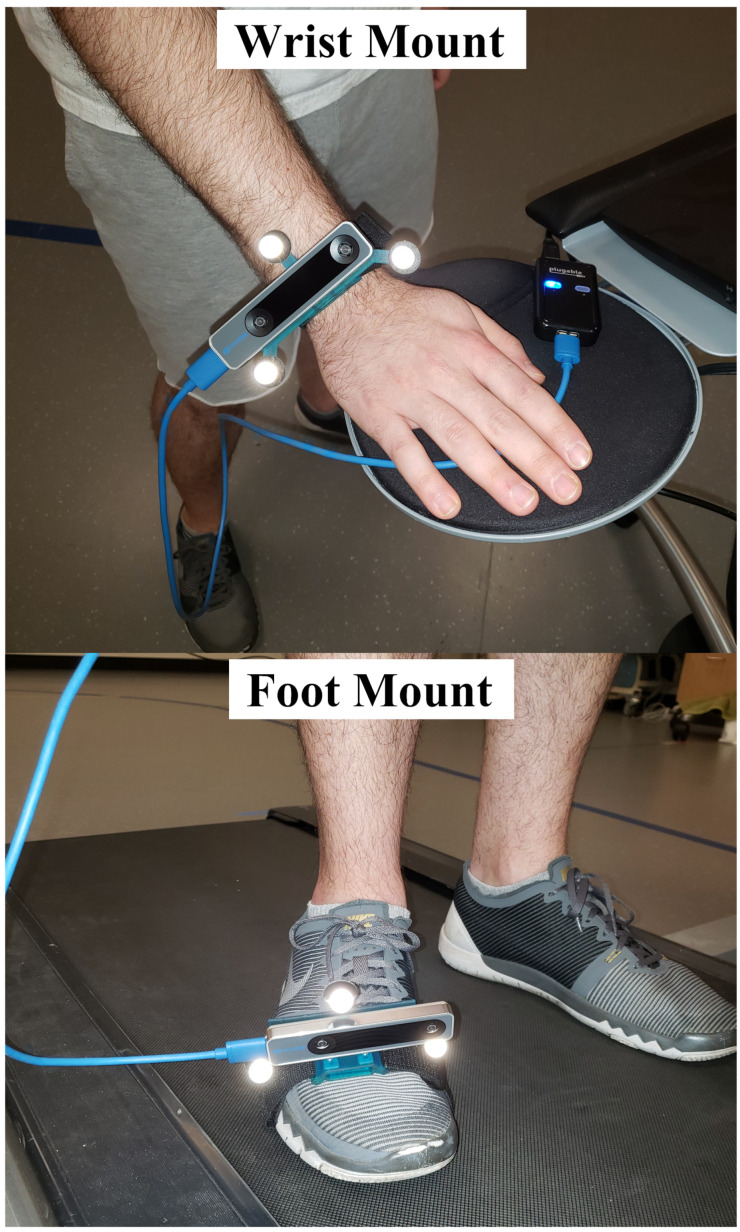
Attached jig fitted with Intel T265 camera and reflective markers.

To localize the T265 camera using the motion capture system we designed and 3D-printed a custom jig to rigidly affix three 12 mm retroreflective markers to the Intel T265 camera. The markers form a triangular pattern with the Intel T265 camera occupying the center of the triangle as shown in Figure 1. Three tracking markers were sufficient to reconstruct an orthogonal coordinate axis representing the jig’s position and orientation in space. This enabled an independent means of tracking the T265 camera’s position and orientation using the optical motion capture system. The jig included slots in the bottom to enable the T265 camera to be attached to subjects with a strap. The strap can be used to attach the jig to the wrist of a human subject similar to the way one attaches a watch. The strap also allows the jig to be attached to the foot or ankle as shown in Figure 2.

### 2.3. Robotic Arm

Although modern motion capture systems provide precise estimates of marker position under ideal conditions, they do not always attain this level of accuracy. For example, the accuracy is reduced during rapid movements, when markers are occluded from one or more cameras, or when the locations of markers are confounded. Most motion analysis systems include software to review and edit the trajectories of individual markers. This enables an expert to more accurately identify the tracks of individual markers and to interpolate trajectories during periods of occlusion. However, the final accuracy then partly depends on the skill of the analyst.

**Figure 3:**
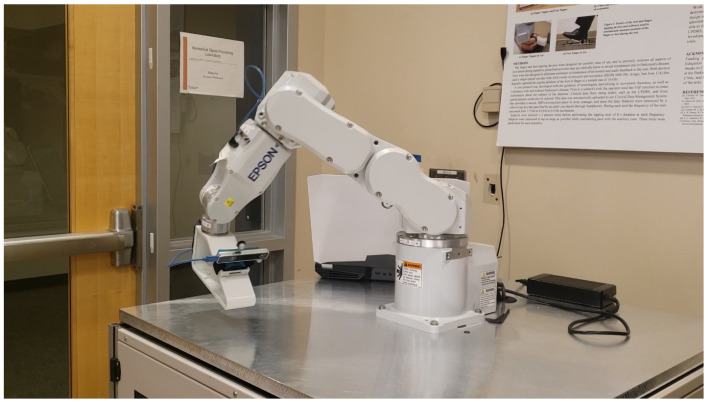
Intel T265 camera attached to Epson C3 robotic arm with custom foot jig.

Because of these limitations, this technology was evaluated with an Epson C3 robotic arm (Epson Robots, Carson, CA, USA) as shown in Figure 3. The Epson C3 has six degrees of freedom, and is capable of movements that are repeatable, precise, and rapid. Although the arm was designed for industrial assembly, it is well suited for controlled studies of movement. The Epson C3 provides maximum angular velocities of each of the six joints in the range of 450–720 °/s, can repeat movements with accuracy of ±0.02 mm, and has a work area of ±0.48 m × ±0.48 m × ±0.48 m. The arm does not suffer from occlusions such as the motion capture system, and it is able to provide accurate position estimates throughout the duration of the recorded movement.

**Figure 4:**
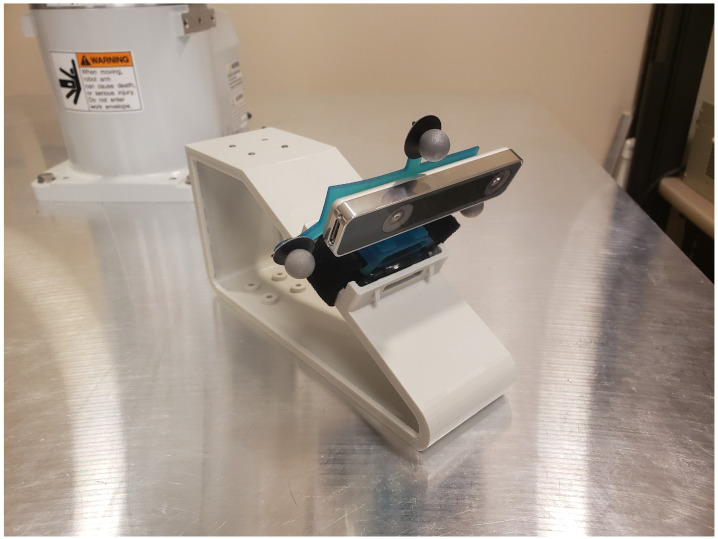
Jig simulating a human foot used to attach the Intel T265 camera to Epson C3 robotic arm.

As with the human subject custom designed jigs enabled the T265 camera to be rigidly connected to the Epson C3. A jig simulating a human foot is shown in Figure 4 and the complete assembly with the robotic arm is shown in Figure 3.

### 2.4. Movement Protocol Design

To evaluate the performance of the T265 camera we chose both rapid movements typical of sports activities and movements typical of clinical assessments for movement disorders. With the T265 camera firmly attached to the wrist of the subject we had him mimic the swing of a baseball bat, a baseball throw, and a finger-to-nose movement. This last movement is commonly used in the assessment of neurological disorders with clinical rating scales [17,18]. With the T265 camera attached to the top of the foot, the subject also performed a 1 min walk on a treadmill. All four of these tasks were performed twice by a healthy male subject.

The human movements were replicated with the Epson C3 robotic arm, which was programmed to simulate a baseball throw, a baseball swing and a human walking. In order to replicate a human walk with the robot arm as accurately as possible we used an actual human walk recording captured using the optical motion capture system. The human walk was first recorded in the motion capture lab using markers placed on the foot. The recorded marker data were then used to calculate the exact orientation and position of the foot. The orientation and position data were then imported into the robot arm control software enabling us to replicate the exact movement with the robot arm. The finger-to-nose movement included a range of motion that was beyond the capability of the robot arm. Instead we developed a zig-zag movement which had similar characteristics.

All movements were repeated four times by the robot arm. Two trials were first collected at slow speed followed by two trials at high speed. The speed was varied to see if motion blur introduced at high velocities would effect the tracking performance accuracy of the T265 camera.

The localization accuracy of the T265 camera can be improved by building a map of the environment prior to data collection during movements of interest. Prior to the first trial the camera was moved throughout the study space over a wide range of angles so that the algorithm in the camera could build a comprehensive map. This only needs to be performed once for a fixed environment and is similar to the calibration process with reflective markers attached to wands used for optical motion capture systems.

### 2.5. Synchronization

Since the motion capture system, robotic arm, and Intel T265 camera operate independently we first had to synchronize their outputs in time. We calculated the lag between the three systems by performing a cross-correlation analysis on the magnitude of their velocity signals. The velocity magnitudes are independent of differences in orientation and initial position, so this permitted us to synchronize the recordings before aligning the reference frames. The velocity of the T265 camera and robotic arm was calculated by taking the derivative of their position outputs. The velocity of the jig was calculated by first finding its centroid using the three reflective markers and then taking the derivative of the centeroid’s position to determine its velocity. We then calculated the cross-correlation of the velocity magnitudes

(1) $$r\left(\ell \right)=\sum_{n=0}^{N-1-\ell }v_{c}\left(n\right)v_{j}\left(n-\ell \right)$$

where $v_{c}$ is the velocity magnitude of the T265 camera, $v_{j}$ is the velocity magnitude of the jig, *N* is the total number of samples, and *l* is the lag between the signals in samples. The fixed lag between the signals was then estimated as

(2) $$\ell_{\mathrm{opt}}=\underset{\ell }{arg max}r\left(\ell \right)$$

The same process was repeated to synchronize the T265 camera with the robotic arm by replacing $v_{c}$ with $v_{r}$ the velocity of the robot arm in the equation above.

### 2.6. Reference Frame Alignment

The T265 camera, optical motion capture system, and robotic arm use different reference frames. The T265 camera estimates the position and orientation of the camera relative to its map of the environment. The optical motion capture system estimates the position of the reflective markers relative to a lab reference frame that is defined by a reference jig that is stationary throughout the recording. The robotic arm defines a reference frame centered at its base.

To calculate measure of performance a common reference frame is needed between the three systems. When comparing the estimates of the T265 camera and the optical motion capture system, we chose to use the reference frame of the optical motion capture system as the common frame of reference. Likewise, when comparing the estimates of the T265 camera with the robotic arm, we chose to use the robotic arm’s reference frame as the common frame of reference.

To perform the reference frame transformation we used a still period of approximately 5 s at the beginning of each recording. In the motion analysis lab, the subject stood as still as possible during this period. Likewise the robotic arm was programmed to briefly remain station before a trial. By design the coordinate axis of the camera is equal to the coordinate axis of the jigs that are used to attach it to the test subject or robotic arm. This is shown in Figure 1 for the jig used in the motion capture lab. Using this relationship we can find a transformation that will transform the T265 camera’s output into the lab reference frame.

### 2.7. Performance Measures

We calculated the Euclidean distance between the T265 camera estimate and the reference position as our performance measure for position. We also calculated the total angular error, which is the angle of rotation required to rotate the T265 camera estimated orientation to the reference orientation, as our performance measure for orientation.

## 3. Results

**Figure 5:**
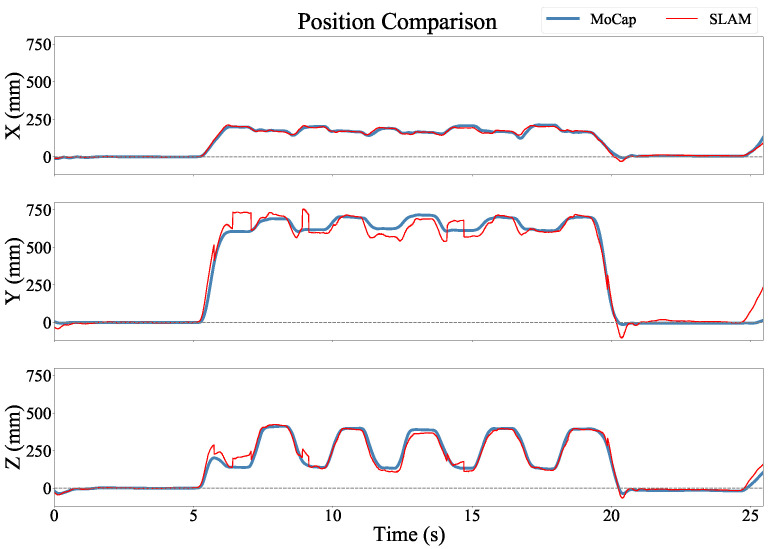
Position versus time for each axis in the Optical Motion Analysis Lab coordinate frame. The two estimates were in strong agreement, but the SLAM estimate included discontinuities.

**Figure 6:**
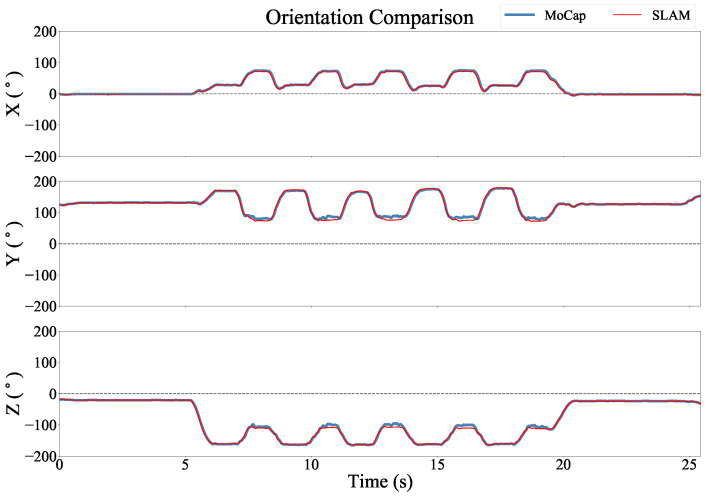
Euler angles versus time for each axis in the Optical Motion Analysis Lab coordinate frame.

Figure 5 shows an example of the position estimates of the two systems during a finger-to-nose trial. While there is excellent overall agreement between the systems, the T265 estimate contains some abrupt changes in the position estimate. This is most visible along the *y* axis. This is most likely caused by the V-SLAM algorithm switching between different configurations of reference points used to estimate the position. Figure 6 shows an example of the estimated Euler angles for the same trial. The discontinuities observed in the position estimate were not discernible in the orientation estimates.

**Figure 7:**
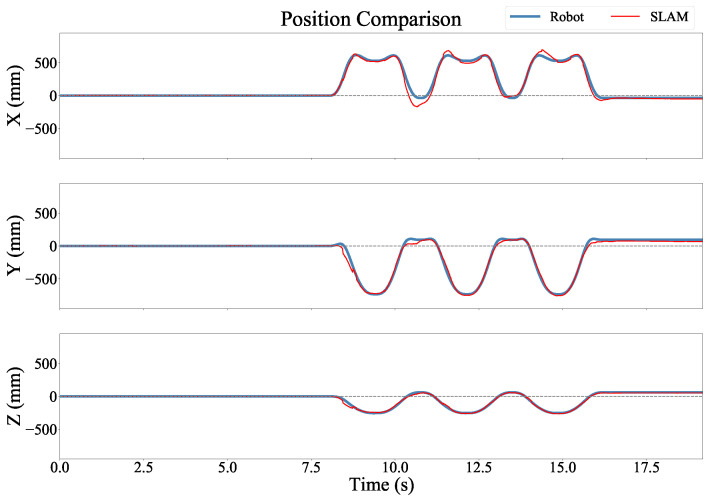
Position versus time for each axis in the Robot Arm coordinate frame. The two estimates were in strong agreement, but the SLAM estimate included discontinuities.

**Figure 8:**
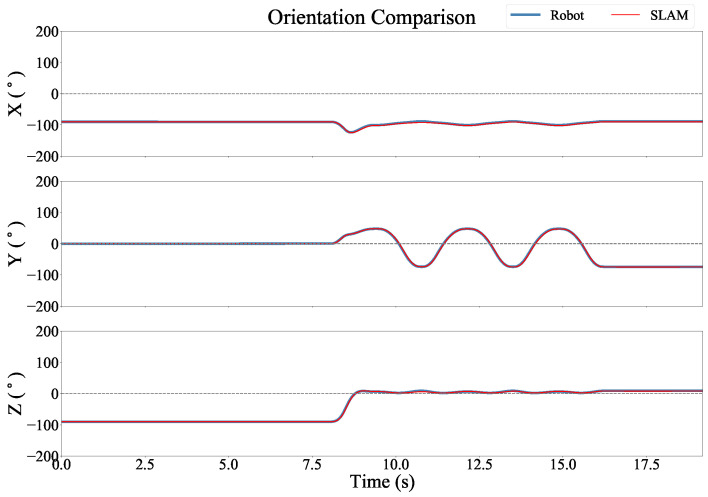
Euler angles versus time for each axis in the Robot Arm coordinate frame.

Figure 7 shows an example of the position estimates as compared to the robotic arm, and Figure 8 shows the orientation estimates as compared to the robotic arm for the same trial. In these recordings the position and orientation estimates were smooth and accurate throughout the trial.

Table 1 lists the average Euclidean and orientation errors for the position and orientation across both trials of each of the seven movement trajectories of the robot arm. Similarly, Table 2 lists the average Euclidean and orientation errors of the position and orientation across both trials for each of the four movements performed by the subject as compared to the motion analysis system.

## 4. Discussion

### 4.1. Performance

The primary aim of this paper was to determine how accurately a modern SLAM system, the Intel T265 tracking camera, can track human movement. Overall there was very good agreement with the motion capture system. Across all eight of the trials the largest angular difference was 4.80° and the largest position difference was 14.6 cm. There was much stronger agreement with the robotic arm. Across all 14 trials the largest angular difference was 1.10° and the largest position difference was 3.37 cm.

The differences in performance as compared to these two standards may be partly due to the larger room with high ceilings in the Motion Analysis laboratory. Since the activity was performed in a relatively small space relative to the volume of the room, the SLAM system may have had difficulty accurately estimating the range of the camera to the anchor points. As different anchor points came in and out of view of the camera and were accepted by the algorithm as valid, this may have caused large changes in the position estimate, as illustrated in Figure 5. We also expect that the Optical Motion Capture system was a less accurate reference standard than the robotic arm, and especially during rapid movements in which the optical markers may have been blurred or temporarily occluded from some of the cameras.

This study also demonstrates some of the limitations of SLAM systems. The algorithms are complicated and include many design parameters. Optimization of the design and parameters tuned for one application, such as augmented reality of a headset, is unlikely to produce the best solution for other applications, such as tracking the position and orientation of the foot during gait. In our results the T265 camera was the most inaccurate during the two rapid movements. It is possible that the algorithm could be tuned to improve performance during rapid movements, and particularly with fusion of the inertial measurement unit.

There was also no indication of when the estimated location would go through an abrupt transition, as shown in the example in Figure 5. This could be a significant limitation in studies where velocities, accelerations, or smoothness of movement are of primary interest.

The performance of SLAM systems is also dependent on the surrounding environment. Environments with many moving elements such as water or trees on a windy day will generally result in worse performance. Similarly, a static background is located far away from the camera, a background with repeated patterns, poor illumination, and a background without distinguishing features that can serve as visual anchor points will also generally worsen performance.

Unlike inertial sensors, cameras must have a free line of sight to a good portion of the environment. Wearable cameras cannot be worn underneath clothing or attached to the body at locations where they are occluded. However, cameras that include inertial sensors should generally perform no worse than inertial sensors alone. Fused solutions should work much better than either technology alone because SLAM generally works well during slow movement and inertial sensors work well during rapid movements when the camera images may contain motion blur.

### 4.2. Considerations for Future Development

The T265 camera used in this study is representative of the current state of the art, but it is not well suited as a wearable for tracking human kinematics. A commercial system based on wearable cameras would require a smaller and lighter form factor with sufficient battery life and wireless synchronization, similar to many wearable inertial sensors.

In many applications tracking human kinematics it will be necessary to record more than one body segment simultaneously. In order to know where two cameras are located within a single environment, the devices will need to share a common map. This might be trivial if the recordings are stored and processed at a later time, as is common with consumer-grade wearable cameras today. Subject to the appropriate data protection controls, the devices will need to communicate wirelessly to maintain time sychronization and to share a common map of the environment.

### 4.3. Privacy and Data Protection Compliance

The processing of personal data through video devices raises privacy issues. That said, the potential privacy risks can be legally addressed by providing appropriate data protection safeguards. These safeguards vary substantially depending on the specific deployment and use case of the system. Accordingly, data controllers deploying the system need to conduct a privacy impact assessment (PIA) for the particular use case. For instance, in some deployments the use of system is substantially equivalent to (1) a video camera integrated in a car for providing parking assistance configured so that it does not collect information related to natural persons (e.g., license plates), (2) a tourist recording videos using a smartphone for personal and family use, or (3) a mountain-biker recording a descent with an actioncam which may include the faces of other mountain-bikers for personal use, and the General Data Protection Regulation (GDPR) does not apply pursuant to Art. 2(2)(c) GDPR (This interpretation has been confirmed by the European Data Protection Board (EDPB) Guidelines on processing of personal data through video devices (EDPB Guidelines 3/2019 Adopted on Plenary Meeting 9–10 July 2019)). In such cases the results of the PIA will indicate that the use-case is out of the scope of the GDPR (or comparable data protection regimes) and compliance is straightforward.

However, since the personal and household exception is narrowly construed, for many deployments of the system the PIA will indicate that the processing is within scope of the relevant data protection regulations. As an example, the use of the system in clinical research, clinical trials, and clinical practice will generally be subject to the GDPR provided the processing is within its broad material and territorial scope (Art. 2 & 3 GDPR). In these cases the PIA should conclude that the processing needs to be in compliance with GDPR and must document, *inter alia*, (1) the specific legal basis for processing (Art. 6 & 9 GDPR), (2) the approach to meet the obligations of the controllers and processors (Art. 24–43 GDPR) including the technical and organizational measures to ensure security of processing (Art. 32 GDPR), (3) the approach to ensure that subjects can exercise their data subject rights (Art. 12–23 GDPR) including using a layered approach to meet the transparency and information obligations, (4) the legal basis and legal instrument for any cross-border transfers of personal data, and (5) the need to conduct a data protection impact assessment (DPIA) pursuant to Art. 35 GDPR.

For controllers and processors that have implemented a comprehensive information security and privacy management system (ISMS/PIMS) such as the ISO 27001/27701 the burden and cost of data protection compliance for deploying such system should be minimal. Furthermore, in the case of deployments of SLAM-based systems in clinical trials for the purpose of endpoint collection, the processing operations are expressly required by the Clinical Trials Regulation (CTR) to ensure the reliability and safety of medical products, and key parts of the PIA analysis have already been established. For instance, the legal basis of processing falls within “processing is necessary for compliance with a legal obligation to which the controller is subject" (Art. 6(1)(c) GDPR) and “processing is necessary for reasons of public interest in the area of public health, such as [...] ensuring high standards of quality and safety of health care and of medicinal products or medical devices" (Art. 9(2)(i) GDPR). However, in the case of processing operations purely related to research activities the legal basis cannot be derived from a legal obligation. Instead, depending on the specific data processing, the legal basis for research may fall under the data subject’s explicit consent (Art. 6(1)(a) GDPR and Art. 9(2)(a) GDPR) or public interest (Art. 6(1)(e) and Art. 9(2)(i) GDPR).

In general, the compliance with these legal requirements is greatly simplified by embedding the overarching principles of personal data processing (Art. 5 GDPR) such as data minimization (i.e., collect the minimum amount of data necessary for the indented purposes), transparency (e.g., providing layered privacy notices and information including warning signs of ongoing video recordings if needed for the particular use case), storage limitations (e.g., ensuring the video data are not stored longer than necessary for the intended purposes), and confidentiality (e.g., implementing security controls proportional to the privacy risks such as data pseudonymization and encryption of personal data to ensure confidentiality through with state-of-the-art security) as part of the data protection by design and default (Art. 25 GDPR) of SLAM-based system deployments.

Some of these principles can be readily achieved by extracting the features necessary to identify anchor points early in the processing. Once these points are detected within a frame, the video frame can be discarded and does not need to be stored (e.g., storage limitation and data minimization). This discards subject-identifying information such as faces that may have been within view of some of the cameras. This is an example of data protection by design that reduces storage requirements, improves battery life, and preserves privacy since the points cannot be used to identify individuals.

### 4.4. Study Limitations

There were several limitations to this study. We only assessed the performance with one subject performing just four tasks. However, the range of tasks included both movement with rapid changes in position and orientation as well as slower movements typical of studies including people with movement disorders.

We also did not use this technology to evaluate the kinematics, which requires determining the alignment between each sensor and the body segment to which it is attached and using two adjoining body segments to estimate joint angles. However, the known constraints in how body segments are interconnected may overcome these limitations. For example, the upper and lower leg are connected by a joint at the knee that can be modeled as a hinge joint with a single degree of freedom. Because of the constraints in the way these two segments are connected, it may be possible to more accurately estimate the knee angle than it is to estimate the orientation of either the upper or lower leg independently.

## 5. Conclusions

Our results demonstrate that the current off-the-shelf SLAM solutions have the potential to be used for human movement tracking. These systems have become much more accurate and easier to use recently, but they still rely on the cameras being able to accurately identify fixed landmarks within the surrounding environment. This technology is a viable alternative to motion analysis systems for precise tracking of human kinematics.

## Figures and Tables

**Table 1:** Position (Euclidan distance) and Orientation errors as compared to the robotic arm.

Trial	Position (mm)	Orientation (${}^{\circ }$)
Zig-Zag Slow 1	4.01	0.50
Zig-Zag Slow 2	3.18	0.36
Zig-Zag Fast 1	3.90	0.29
Zig-Zag Fast 2	3.02	0.30
Swing Slow 1	27.60	1.02
Swing Slow 2	29.05	1.10
Swing Fast 1	27.00	1.02
Swing Fast 2	33.69	0.98
Throw Slow 1	2.04	0.18
Throw Slow 2	2.16	0.18
Throw Fast 1	2.50	0.18
Throw Fast 2	2.24	0.18
Walk 1	14.91	0.58
Walk 2	16.16	0.55

**Table 2:** Position (Euclidan distance) and Orientation errors as compared to the optical motion capture system.

Trial	Position (mm)	Orientation (${}^{\circ }$)
Finger to Nose 1	24.83	2.24
Finger to Nose 2	35.55	3.02
Baseball Swing 1	119.61	3.53
Baseball Swing 2	105.41	4.80
Baseball Throw 1	72.25	3.54
Baseball Throw 2	146.14	3.21
Treadmill Walk 1	13.86	1.83
Treadmill Walk 2	19.02	1.59

## Data Availability

Not applicable.

## References

[1] Durrant-Whyte H., Bailey T. (2006). Simultaneous Localization and Mapping: Part I. IEEE Robot. Autom. Mag..

[2] Rosen D.M., Doherty K.J., Terán Espinoza A., Leonard J.J. (2021). Advances in Inference and Representation for Simultaneous Localization and Mapping. Annu. Rev. Control Robot. Auton. Syst..

[3] Bresson G., Alsayed Z., Yu L., Glaser S. (2017). Simultaneous Localization and Mapping: A Survey of Current Trends in Autonomous Driving. IEEE Trans. Intell. Veh..

[4] Mur-Artal R., Tardós J.D. (2017). ORB-SLAM2: An Open-Source SLAM System for Monocular, Stereo, and RGB-D Cameras. IEEE Trans. Robot..

[5] Moeslund T.B., Hilton A., Krüger V. (2006). A survey of advances in vision-based human motion capture and analysis. Comput. Vis. Image Underst..

[6] Sabatini A.M. (2011). Estimating three-dimensional orientation of human body parts by inertial/magnetic sensing. Sensors.

[7] Li X., Cao J., Li H., Yu P., Fan Y., Xiao Y., Yin Y., Zhao X., Wang Z.L., Zhu G. (2021). Differentiation of Multiple Mechanical Stimuli by a Flexible Sensor Using a Dual-Interdigital-Electrode Layout for Bodily Kinesthetic Identification. ACS Appl. Mater. Interfaces.

[8] Shiratori T., Park H.S., Sigal L., Sheikh Y., Hodgins J.K. (2011). Motion Capture from Body-Mounted Cameras. ACM Trans. Graph..

[9] Pacher L., Chatellier C., Vauzelle R., Fradet L. (2020). Sensor-to-segment calibration methodologies for lower-body kinematic analysis with inertial sensors: A systematic review. Sensors.

[10] Kuo J., Muglikar M., Zhang Z., Scaramuzza D. (2020). Redesigning SLAM for arbitrary multi-camera systems. arXiv.

[11] Concha A., Loianno G., Kumar V., Civera J. Visual-inertial direct SLAM. Proceedings of the 2016 IEEE International Conference on Robotics and Automation (ICRA).

[12] Ye X., Ji X., Sun B., Chen S., Wang Z., Li H. (2020). DRM-SLAM: Towards dense reconstruction of monocular SLAM with scene depth fusion. Neurocomputing.

[13] Debeunne C., Vivet D. (2020). A review of visual-LiDAR fusion based simultaneous localization and mapping. Sensors.

[14] Potirakis S.M., Servières M., Renaudin V., Dupuis A., Antigny N. (2021). Visual and Visual-Inertial SLAM: State of the Art, Classification, and Experimental Benchmarking. J. Sens..

[15] Mur-Artal R., Montiel J.M.M., Tardós J.D. (2015). ORB-SLAM: A Versatile and Accurate Monocular SLAM System. IEEE Trans. Robot..

[16] Aurand A.M., Dufour J.S., Marras W.S. (2017). Accuracy map of an optical motion capture system with 42 or 21 cameras in a large measurement volume. J. Biomech..

[17] Schmitz-Hubsch T., du Montcel S.T., Baliko L., Berciano J., Boesch S., Depondt C., Giunti P., Globas C., Infante J., Kang J.S. (2006). Scale for the assessment and rating of ataxia: Development of a new clinical scale. Neurology.

[18] Goetz C.G., Tilley B.C., Shaftman S.R., Stebbins G.T., Fahn S., Martinez-Martin P., Poewe W., Sampaio C., Stern M.B., Dodel R. (2008). Movement Disorder Society-sponsored revision of the Unified Parkinson’s Disease Rating Scale (MDS-UPDRS): Scale presentation and clinimetric testing results. Mov. Disord..

